# Love as Calculation: The Internalized Rationality of Digital Intimacy and the Erosion of Social Flow

**DOI:** 10.3390/bs16091649

**Published:** 2026-09-14

**Authors:** Jiaxin Jiang, Chunli Ji, Tian Zhao, Erose Sthapit

**Affiliations:** 1 Centre for Gaming and Tourism Studies, Macao Polytechnic University, Macao 999078, China; p2413923@mpu.edu.mo; 2 Department of Marketing, Retail and Tourism, Manchester Metropolitan University, Manchester M15 6BH, UK; e.sthapit@mmu.ac.uk

**Keywords:** calculative subjectivity, social flow, partner phubbing, regret aversion, neoliberalism

## Abstract

As neoliberal rationality permeates private life, romantic relationships are increasingly managed as calculated projects rather than spontaneous, autotelic bonds. Drawing on Self-Determination Theory (SDT), this study examines how calculative subjectivity is associated with diminished social flow—the state of dyadic synchrony—within intimate relationships in the context of contemporary societal involution, a state of hyper-competition and intense social comparison. Analyzing data from 402 Chinese digital natives via PLS-SEM, we reveal that calculative subjectivity demonstrates a substantial negative association with social flow, statistically mediated through internalized cognitive rationalities (regret aversion, sunk cost sensitivity, self-monitoring) and external behavioral distractions (partner phubbing). Specifically, the “market-in-mind” correlates with a substantial cognitive barrier alongside the physical interference of the “device-in-hand.” These findings are theoretically consistent with the philosophical critique regarding the “agony of eros,” suggesting that a prominent barrier to authentic intimacy in the algorithmic age is not merely technological mediation, but the internalized instrumental rationality that colonizes the human mind.

## 1. Introduction

With the development of digital platforms and algorithmic matchmaking, contemporary intimacy embodies a profound contradiction. The experience of deep, enduring bonding has become increasingly elusive. The digital architecture of modern romance, designed to maximize efficiency, has arguably produced the opposite effect. Rather than facilitating better choices, the algorithmic abundance of potential partners has precipitated a state of cognitive overload and decision paralysis (Apostolou et al., 2024; Pronk & Denissen, 2020). Consequently, the technological promise of liberation has transformed into a cycle of inescapable decision fatigue, where the sheer volume of “swiping” correlates negatively with the likelihood of forming meaningful relationships (Thomas et al., 2022).

This behavioral shift signals a profound transformation in the ontology of love. Modern interpersonal bonds have acquired a “liquid” fragility, characterized by transience and a pervasive fear of commitment (Bauman, 2013). This fragility is not merely an emotional deficit but the result of the thorough rationalization of intimacy, or “emotional capitalism,” where the search for a partner is subconsciously reconstructed as a market transaction requiring continuous auditing and optimization (Illouz, 2012). Under this pressure for self-optimization, the romantic experience is substantively eroded, leading to a systemic “agony of eros” (Han, 2017). In this environment, the capacity to inhabit the present moment is sacrificed for a defensive, calculative posture against future uncertainty (D’Angelo & Toma, 2017), often exacerbated by the commodification of desire under neoliberal logics (Lo, 2024).

The internalization of neoliberal governmentality serves as the underlying structural logic associated with this disconnection, reshaping the romantic self into an entrepreneur of the self (Foucault, 2010). Crucially, within the socio-cultural context of China—where the present study’s sample is situated—this neoliberal logic manifests specifically as a pervasive phenomenon known as “involution” (*neijuan*). Originally an anthropological concept describing a system that complicates itself internally without meaningful growth, contemporary involution has evolved to denote a societal state of hyper-competition, relentless social comparison, and intense anxiety over diminishing returns (W. Zhang et al., 2024; Y. Zhang & Ji, 2023). This structural pressure has inevitably spilled over from the educational and professional arenas into the dating market, where Chinese platform economics commodify romance and exacerbate mating anxieties (Chan, 2020; M. Wang, 2025). In a highly involuted society, young individuals often face precarious futures, forcing them to adopt a defensive posture that maximizes their “return on investment” (ROI) across all life domains, including romance. This involuted rise to a Calculative Subjectivity, a construct reflecting a psychological framework where intimacy is viewed not as an autotelic bond, but as a project of risk management and return on investment (Ball & Olmedo, 2023), intertwining personal worth with market-driven metrics of success (Jung, 2024). As individuals become preoccupied with maintaining their market value and hedging against bad investments, the unconditional attention required for the Other erodes (Han, 2017). Although macro-level scholarship has illuminated how neoliberal structures shape digital dating platforms (e.g., Illouz, 2012; Lo, 2024), the specific cognitive mechanisms and how this macro-logic infiltrates the micro-moments of a date remain underexplored. Specifically, it lacks an understanding of how this mindset disrupts Social Flow, the shared state of absorption and dyadic synchrony that forms the bedrock of connection (Walker, 2010), and which empirical studies link to improved relational outcomes such as trust and emotional resilience (Hackert et al., 2023). This oversight is particularly pressing amid rising loneliness epidemics, with recent surveys indicating that over 20% of adults in the U.S. and increasingly in hyper-competitive Asian societies experience chronic loneliness, frequently attributing it to technology’s fragmentation of social bonds (Batanova et al., 2024).

Crucially, this psychological calculation is inextricably linked to the device in hand. Within the logic of surveillance capitalism, the smartphone is a distraction that constantly harvests attention and offers alternative options (Cohen, 2019), amplifying systemic vulnerabilities through pervasive data extraction and behavioral prediction (Curran, 2023). Yet, the relationship between the internal calculative mind and the external phubbing behavior (partner phone snubbing) remains ambiguous, with recent meta-analyses highlighting its detrimental effects on relational satisfaction and conflict (Ni et al., 2025).

To address these gaps, this study employs Self-Determination Theory (SDT) as an overarching theoretical frame to explore how calculative subjectivity inhibits social flow through four distinct pathways: the cognitive burdens of regret aversion and sunk cost sensitivity, the defensive performance of self-monitoring, and the physical interference of partner phubbing. Unlike the traditional relational literature that relies on generalized affective traits (e.g., attachment anxiety (Mikulincer & Shaver, 2010)) or diffuse cognitive anxieties (e.g., FOMO (Przybylski et al., 2013)), these four specific mediators are deliberately selected to capture the precise “investment and return” ethos of neoliberal market logic, bridging macro-level calculative mindsets with micro-level dyadic disruption.

By quantifying these mechanisms, this research aims to answer a fundamental question of the algorithmic age: Is the true barrier to intimacy the technological device in hand, or the instrumental rationality that has colonized our minds? To what extent does calculative subjectivity disrupt social flow via these pathways, and does internal rationality outweigh technological factors in eroding relational depth? Leveraging a diverse sample of digital natives, our empirical approach not only tests these pathways but also utilizes SDT as an interpretive lens to offer actionable insights for mitigating neoliberal influences on relational well-being.

## 2. Literature Review and Hypotheses Development

### 2.1. Self-Determination Theory

Self-Determination Theory (SDT) posits that high-quality intimate relationships rely on satisfying three basic psychological needs: autonomy, competence, and relatedness (Ryan, 2017). However, contemporary sociological and psychological research indicates that neoliberal ideology fundamentally thwarts these needs. Operating as a cultural ideology of competition and market logic (Ginn et al., 2022), neoliberalism exacerbates extrinsic orientations where self-worth depends on external validations, thereby displacing intrinsic motivation (Kasser & Ryan, 1996) and commodifying emotions (Lo, 2024).

Rooted in ontological insecurity and precarious futures (del Río, 2026), which in the Chinese context are profoundly amplified by societal involution (M. Wang, 2025), this outward-seeking orientation is closely associated with the instrumentalization of intimacy as a risk-management tool. Under the cultural specificities of an involuted society, the dating market is navigated with the same zero-sum anxiety as the hyper-competitive labor market and commercialized platform economics (Chan, 2020), severely frustrating the psychological need for relational autonomy. Consequently, others are increasingly viewed as competitors or strategic assets rather than authentic companions, fostering social disconnection (Becker et al., 2021). Within intimate relationships, pursuing extrinsic goals—such as image or wealth—directly diminishes satisfaction by frustrating basic psychological needs (Leung & Law, 2019). For instance, linking self-esteem to economic success heightens relational conflict and weakens autonomy support (D. E. Ward et al., 2021), while materialistic values universally impair relational well-being (Unanue et al., 2014).

Overall, SDT provides a unifying conceptual framework for this research. Rather than empirically testing the specific pathways of basic need frustration, this study utilizes SDT as an interpretive lens to illustrate how neoliberal-driven and involution-amplified calculative subjectivity conceptually threatens fundamental psychological needs, thereby concurrently activating four parallel cognitive (regret aversion, sunk cost sensitivity), performative (self-monitoring), and behavioral (partner phubbing) mediators.

### 2.2. Calculative Subjectivity and Social Flow

To bridge macro-level neoliberal influences and micro-level relational dynamics, calculative subjectivity is proposed as a second-order construct comprising extrinsic merit and performance orientation. Integrating sociological critiques of the entrepreneurial self (Ball & Olmedo, 2023; Foucault, 2010) with Self-Determination Theory (SDT) (Kasser & Ryan, 1996; Ryan et al., 2021), this construct represents a psychological mechanism through which neoliberal ideology and hyper-competitive cultural pressures thwart basic needs and disrupt intimacy.

Extrinsic merit involves anchoring partner desirability and self-worth in quantifiable external markers like status and wealth (Fletcher et al., 2004). In an involuted dating market, where individuals are often implicitly evaluated as social commodities, this tendency is severely amplified. This materialistic orientation promotes objectifying standards that erode interpersonal closeness (Moldes, 2025). Performance orientation reflects instrumental rationality, framing relationships as optimizable projects geared toward measurable returns (X. Wang et al., 2022). For digital natives navigating a precarious, zero-sum societal environment, this mindset functions as a psychological defense mechanism; they apply strict cost–benefit analyses to romantic investments to avoid the devastating penalty of “wasting” limited emotional resources. Such mindsets predict declining relationship satisfaction and maladaptive conflict communication (Park et al., 2026).

Conversely, the dependent variable, social flow, differs from individual flow (Csikszentmihalyi & Csikszentmihalyi, 1992). It constitutes a dyadic state of shared absorption, mutual synchrony, and effortless coordination (Hackert et al., 2023; Walker, 2010) that buffers relational stress (Capozzi, 2025). Within SDT, flow’s autotelic nature relies on intrinsic motivation and satisfied psychological needs, particularly relatedness (Ryan, 2017). When individuals adopt the instrumental, future-oriented posture of calculative subjectivity, their focus shifts from present-centered engagement to cost–benefit analysis and external validation. This systematically undermines the intrinsic motivation and genuine relatedness required for dyadic synchrony (Hackert et al., 2023; Ryan et al., 2021). By thwarting need satisfaction at the micro-interactional level, calculative subjectivity disrupts the relational presence and mutual attunement central to social flow. Therefore, we propose:

**H1.**  *Calculative subjectivity is negatively associated with social flow in intimate relationships.*

### 2.3. The Mediating Role of Cognitive Burdens: Regret and Sunk Cost Sensitivity

A defining feature of calculative subjectivity is the tendency toward relational maximization, wherein individuals pursue not merely satisfactory but ostensibly optimal relational outcomes (Mikkelson & Ray, 2020). This orientation is particularly pronounced in digitally mediated and highly involuted environments marked by perceived abundance of alternatives and minimal switching costs, where ongoing comparison with hypothetical superior options becomes routine (Pronk & Denissen, 2020; Thomas et al., 2022). Individuals exhibiting maximization tendencies often adopt a rejection-focused decision strategy that prioritizes negative cues to eliminate options rapidly, thereby diminishing the likelihood of commitment (Mikkelson & Ray, 2020). Even following a commitment, the search for better alternatives persists through counterfactual reflection—“what if another choice had been superior?”—resulting in pronounced post-decisional regret (Ma & Roese, 2014; Roese & Summerville, 2005). Empirical studies on the “paradox of choice” in digital dating demonstrate that a perceived abundance of potential partners often exacerbates choice-related anxiety and regret, ultimately diminishing satisfaction with current romantic choices, as individuals remain psychologically anchored to unrealized alternatives (D’Angelo & Toma, 2017; Finkel et al., 2012).

From a Self-Determination Theory perspective, chronic regret aversion conceptually signals ongoing frustration of the competence need: the individual experiences reduced efficacy and agency amid an overwhelming array of algorithmic possibilities, making confident affirmation of the current choice psychologically burdensome (Ryan et al., 2021). Counterfactual rumination further commandeers cognitive resources that would otherwise support relational immersion and dyadic synchrony. Accordingly, regret aversion is hypothesized to transmit the adverse influence of calculative subjectivity onto social flow by eroding the capacity for undivided, present-focused engagement. Therefore, we propose:

**H2.**  *Regret aversion mediates the negative relationship between calculative subjectivity and social flow.*

Concurrently, calculative subjectivity fosters a retrospective cognitive burden through elevated sensitivity to sunk costs. When relationships are conceptualized as capital investments subject to continuous auditing, previously expended resources—time, emotional labor, financial outlays—serve as salient anchors that encourage irrational persistence (Arkes & Blumer, 1985). Unlike adaptive commitment grounded in expected future benefits, this sensitivity manifests as defensive escalation motivated by the need to justify prior allocations and avert the aversive recognition of loss (Goodfriend & Agnew, 2008; Rego et al., 2018). Empirical findings indicate that greater perceived investment in the past, particularly in time and material resources, markedly increases the probability of remaining in low-satisfaction relationships, even when dissolution appears objectively preferable (Coleman, 2009; Rusbult, 1980). Such persistence is frequently accompanied by effort justification and rumination over irrecoverable expenditures, which further consumes cognitive capacity and redirects attention from the partner toward historical reconciliation (Van Putten et al., 2010; Zentall, 2010). Within Self-Determination Theory, sunk cost sensitivity can be theoretically interpreted as a manifestation of thwarted competence and autonomy: the individual feels compelled to “rescue” the investment to restore perceived efficacy, yet this compulsion erodes autonomous involvement and authentic relatedness in the present (Ryan, 2017). By fixating attention on the past rather than the unfolding interaction, sunk cost sensitivity is hypothesized to serve as a second pathway through which calculative subjectivity impedes social flow. Therefore, we propose:

**H3.**  *Sunk cost sensitivity mediates the negative relationship between calculative subjectivity and social flow.*

### 2.4. The Mediating Role of Self-Monitoring

Calculative subjectivity fosters a heightened need for strategic self-presentation in order to maintain perceived extrinsic merit and avoid relational devaluation in competitive dating environments. This orientation promotes elevated self-monitoring, defined as the ongoing adjustment of self-presentation to situational cues and anticipated social evaluations (Snyder, 1974). In the context of calculative logic and digitally mediated intimacy, self-monitoring shifts from adaptive regulation to a resource-intensive form of defensive impression management that undermines the authenticity and spontaneity required for social flow. Digital dating platforms exacerbate the demand for idealized self-presentation, creating tension between projected desirability and genuine expression (J. Ward, 2017). Individuals frequently engage in strategic enhancement or selective disclosure to optimize matching outcomes and signal market value (Hall et al., 2010).

From a Self-Determination Theory perspective, such behavior reflects controlled motivation governed by external contingencies rather than intrinsic relational interest (Ryan, 2017). The sustained effort devoted to curating and defending an idealized image consumes attentional resources, redirects focus toward self-surveillance, and thwarts autonomy within the relationship (Deci & Ryan, 2000). This performative preoccupation is often accompanied by emotional detachment or limited commitment during interaction, further obstructing deep emotional synchrony and mutual attunement (Overall et al., 2015). Moreover, chronic self-monitoring generates an authenticity paradox: the very tactics used to secure relational advantage diminish perceived genuineness for both self and partner (Wickham & Bond, 2020). Each encounter becomes implicitly structured as a performance that must be successfully executed, transforming spontaneous relational engagement into a high-stakes regulatory task. This dynamic has been conceptualized as affective neoliberal governmentality, wherein emotional regulation and self-presentation are internalized as personal moral imperatives (Smoliak et al., 2025). The resulting self-vigilance suppresses authentic emotional disclosure, inhibits vulnerability, and prevents the mutual openness and co-regulation necessary for dyadic resonance and social flow (Coutinho et al., 2019; Hackert et al., 2023). Similarly, regret aversion and sunk cost sensitivity establish a retrospective and counterfactual cognitive trap, aligning with a perpetual calculus of relational return. Together, these intertwined mechanisms illustrate how the internal market logic is structurally associated with the transition of intimacy from an autotelic sanctuary to an optimized transactional arena. These insights collectively underscore that the erosion of digital romance is fundamentally linked to internalized calculative schemas rather than mere technological artifacts, offering a robust framework for rethinking relational well-being in the digital age. Therefore, we propose:

**H4.**  *Self-monitoring mediates the negative relationship between calculative subjectivity and social flow.*

### 2.5. The Mediating Role of Partner Phubbing

Partner phubbing refers to the behavior of diverting attention to one’s smartphone during a face-to-face interaction, effectively snubbing the present companion (Roberts & David, 2016). In this study, phubbing emerges as more than an incidental distraction, functioning instead as a concrete expression of algorithmic entanglement: the device serves as an interface for ongoing market surveillance, alternative management, and risk mitigation within the logic of surveillance capitalism (Cohen, 2019).

This entanglement is often propelled by fear of missing out, which empirical work consistently links to phubbing behavior (Ansari et al., 2024), particularly in contexts where digital platforms amplify perceptions of alternative opportunities (Elhai et al., 2019). When relationships are framed as capital projects requiring continuous auditing, the smartphone assumes a central role in tracking external opportunities, evaluating comparative value, and alleviating uncertainty about superior alternatives (Schokkenbroek et al., 2022). Further evidence suggests that problematic media use and addiction predict partner phubbing more strongly than personality traits, highlighting phubbing’s ties to structural algorithmic dependence rather than an isolated disposition (Ni et al., 2025). This dependence often escalates into electronic partner surveillance—tracking a partner’s online activity to manage relational threats—which further entrenches device centrality and amplifies phubbing tendencies (Aloia, 2023).

Partner phubbing, in turn, impairs social flow by violating norms of mutual responsiveness and joint attention. Frequent phone-checking during interaction conveys exclusion and devaluation, indicating that digital content takes precedence over the present partner (Roberts & David, 2024). This pattern reliably diminishes perceived responsiveness, intimacy, and communication quality (McDaniel et al., 2021). Unlike cognitive mechanisms, phubbing creates a tangible physical and spatiotemporal barrier: the device’s presence and interruptions fragment the continuous, co-present attention essential for social flow (Hackert et al., 2023; Walker, 2010). From a Self-Determination Theory lens, these violations conceptually thwart relatedness by eroding emotional availability and co-regulation, blocking dyadic synchrony and shared absorption (Ryan et al., 2021). Partner phubbing thus operates as a key behavioral pathway linking calculative subjectivity to reduced social flow. By externalizing calculative logic into the interaction space, the smartphone transforms from a neutral medium into an active agent of disconnection that severs the attentional continuity and relational presence crucial for flow. Accordingly, we propose:

**H5.**  *Partner phubbing mediates the negative relationship between calculative subjectivity and social flow.*

**Figure 1:**
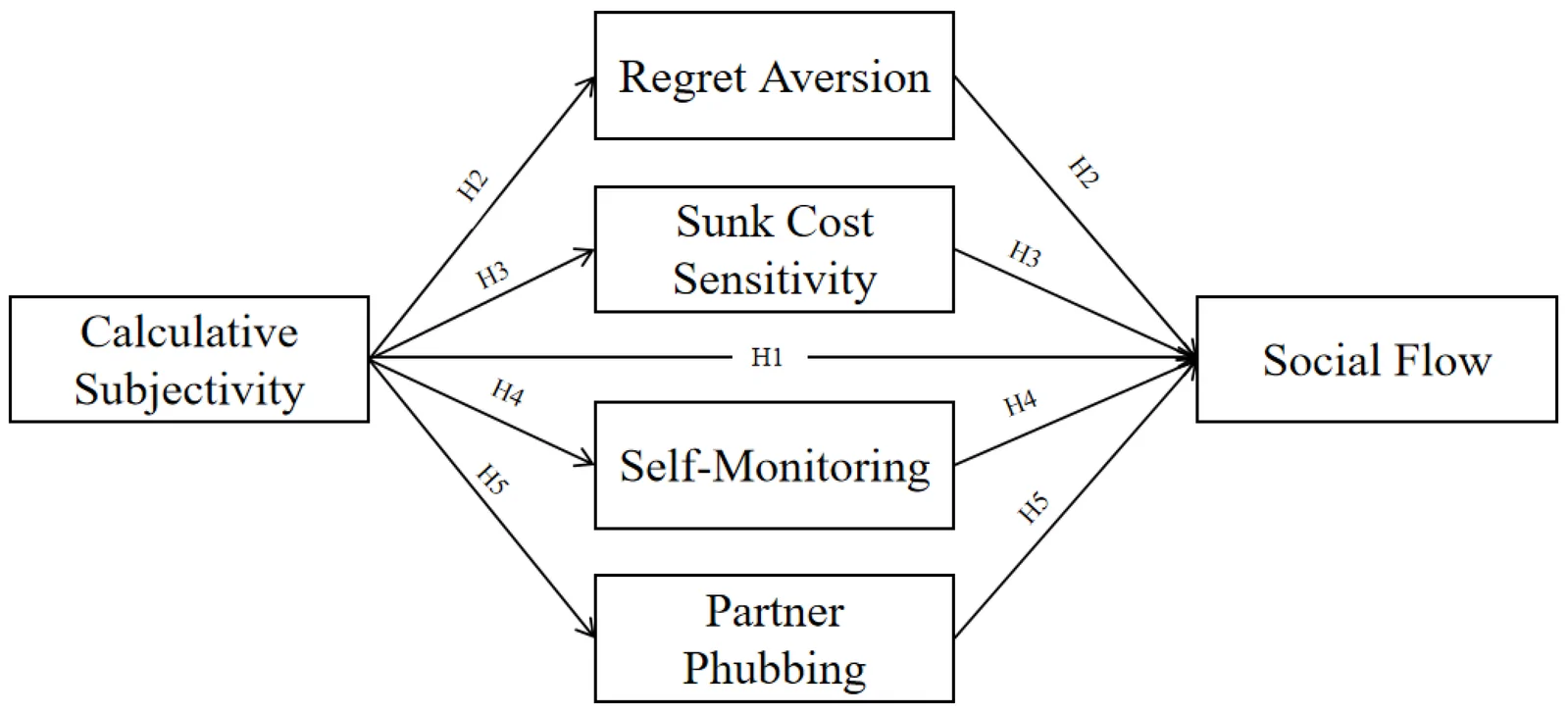
The conceptual model.

Based on the comprehensive literature review and the hypotheses developed above, this study proposes a conceptual research model, as illustrated in Figure 1.

## 3. Methodology

### 3.1. Research Design and Sampling Procedure

A cross-sectional survey design was employed to test the proposed model. This retrospective approach allows for capturing a broad range of relationship experiences, including those from non-partnered individuals, which is essential for examining the enduring effects of calculative subjectivity on social flow (e.g., Joel et al., 2018). The questionnaire was developed as an electronic survey and distributed using the Wenjuanxing online platform (Changsha Ranxing Information Technology Co., Ltd., Changsha, China). Participants were recruited by posting invitations on major Chinese social media platforms, including Weibo, Xiaohongshu, and WeChat groups. To ensure data applicability, specific inclusion criteria were established. Respondents identified as single (not currently dating) or separated were explicitly instructed to reflect upon their most recent significant intimate relationship experience retrospectively. This inclusion of non-partnered individuals is justified by the need to represent the full spectrum of digital-era intimacy, where transient and post-relationship reflections are common. Indeed, established psychological methodologies support the validity of retrospective self-reports in capturing salient subjective experiences, such as the cognitive processing of regret (Gilovich & Medvec, 1995) and the episodic recall of flow states (Jackson & Marsh, 1996).

Participants provided informed consent prior to completing the survey, were assured of anonymity and confidentiality, and had the right to withdraw at any time without penalty. Data were stored securely and used solely for research purposes. A total of 433 questionnaires were initially collected. Following rigorous data cleaning procedures—which involved the exclusion of responses with insufficient completion time, patterned responding, or failure on reverse-coded attention checks—402 valid questionnaires were retained, yielding a robust data-retention rate of 92.84%. An a priori power analysis (G*Power 3.1 (Heinrich Heine University, Düsseldorf, Germany); f^2^ = 0.05, α = 0.05, power = 0.95) indicated a minimum sample requirement of 218, which the final sample of 402 significantly exceeds.

The demographic characteristics of the sample are presented in Table 1. Of the 402 respondents, 57.2% were female (*n* = 230), and 41.0% were male (*n* = 165). Regarding age distribution, the 26–30 age group was the most significant (34.3%), followed by the 18–25 age group (27.9%), indicating a sample predominantly composed of young digital natives. In terms of education, the majority held a bachelor’s degree or higher (86.1%), with 61.0% holding a bachelor’s degree and 20.1% holding a master’s degree. Regarding relationship status, the sample encompassed all five surveyed categories: 31.8% were single (not dating), 12.9% were single (dating), 26.1% were in a relationship, 22.1% were married, and 7.0% were separated or divorced. Overall, the sample is characterized by a high level of education and a younger demographic profile, aligning well with the study’s focus on intimate relationships in the digital era.

### 3.2. Measures

All measurement scales employed in this study were adapted from the established literature. To achieve cultural and linguistic equivalence, the original English scales were translated into Chinese using the standard back-translation procedure followed by pilot testing with 30 respondents and evaluation by a panel of domain experts to refine wording, ensure comprehensibility, and confirm content validity. Crucially, to prevent measurement–construct misalignment, participants were provided with a strict instructional preamble directing them to evaluate all statements specifically within the context of their digital dating and romantic experiences. Unless otherwise specified, all items were assessed using a 7-point Likert scale ranging from 1 (Strongly Disagree) to 7 (Strongly Agree). Full item wording for all scales is provided in Table 2.

To accurately capture the unique theoretical conceptualization of Calculative Subjectivity (CS) within the specific context of digital intimacy, the items for this second-order latent construct were substantially modified from their original sources. CS was modeled with two first-order dimensions: Extrinsic Merit and Performance Orientation. Extrinsic Merit was operationalized using a three-item scale substantially modified from the extrinsic goal domains of the Aspirations Index (Kasser & Ryan, 1996) and the Contingencies of Self-Worth subscales (Crocker et al., 2004) to capture the anchoring of partner desirability and self-worth in quantifiable external markers. Performance Orientation was measured with a three-item scale substantially modified from Vandewalle (1997) to assess the extent to which individuals frame intimate relationships as calculated, outcome-driven projects. Together, the six items index CS as a higher-order propensity to evaluate and manage relationships through market-like, instrumentally quantifiable criteria. The content validity of this synthesized and substantially modified scale was rigorously confirmed by the expert panel during the pilot phase, providing psychometric justification for its use.

In contrast to the CS scale, the remaining constructs were adapted from existing scales with only minor contextual wording changes. The dependent variable, Social Flow (SF), was assessed with a seven-item scale adapted from Walker (2010) to capture interactional immersion and dyadic synchrony. To ensure comprehensive content validity, this scale includes a reverse-coded indicator (“I often keep my guard up…”) explicitly designed to capture vulnerability avoidance. Theoretical foundations of flow emphasize that the “loss of self-consciousness” and the willingness to relinquish defensive psychological boundaries are essential prerequisites for deep dyadic resonance (Walker, 2010). Therefore, this reverse-coded item is theoretically vital for indexing the requisite absence of self-surveillance during flow states. For the mediating mechanisms, Regret Aversion (RA) was measured with a three-item scale adapted from Mikkelson and Ray (2020) to index anxiety and avoidance tendencies in decision contexts. Sunk Cost Sensitivity (SCS) was assessed with a three-item scale developed on the basis of Rego et al. (2018). Self-Monitoring (SM) was measured with four items adapted from Hall et al. (2010) to capture defensive impression-management in digital environments. Finally, Partner Phubbing (PP) was measured with a three-item scale adapted from Roberts and David (2016) to capture the frequency of phone-checking during dyadic interactions.

### 3.3. Common Method Bias

To address potential Common Method Bias (CMB) inherent in single-source, all-self-reported data, this study employed both procedural and statistical remedies (Podsakoff et al., 2003). Procedurally, respondent anonymity and confidentiality were strictly guaranteed to reduce evaluation apprehension. Furthermore, clear instructional preambles were utilized to create psychological separation between the measurement of different constructs. Statistically, a Full Collinearity Assessment was performed during the structural model evaluation. Following Kock (2015)’s recommendation, all VIF values remained below 3.3, indicating that CMB did not pose a significant threat. To provide complementary diagnostic evidence, Harman’s single-factor test was conducted using unrotated principal component analysis. The results revealed that the first major factor accounted for 31.78% of the total variance, well below the critical threshold of 50%. Collectively, these procedural and statistical diagnostics suggest that CMB is unlikely to confound the results of this study.

## 4. Data Analysis Strategy and Results

Data were analyzed using SmartPLS 4.1.6. Prior to executing the analytical steps, the methodological suitability of PLS-SEM for our theoretical framework must be highlighted. While traditionally viewed primarily as a predictive tool, recent methodological advancements have firmly established PLS-SEM as a robust method for explanatory research and rigorous theory testing (Hair et al., 2021). PLS-SEM was explicitly chosen for this study for two critical reasons. First, our research model is highly complex, incorporating a second-order latent construct (Calculative Subjectivity) alongside four parallel mediators; PLS-SEM efficiently estimates such complex structural models without imposing restrictive data distributional assumptions (Hair et al., 2021). Second, regarding mediation testing, the recent literature validates that PLS-SEM, coupled with advanced non-parametric bootstrapping techniques, offers superior statistical power for testing complex, multiple-parallel mediation pathways compared to traditional covariance-based approaches (Nitzl et al., 2016).

Following the two-stage PLS-SEM evaluation procedure recommended by Hair et al. (2021), the analysis involved: (1) assessment of the measurement model, including evaluations of indicator reliability, internal consistency reliability, convergent validity, and discriminant validity; and (2) assessment of the structural model after satisfactory measurement model performance. Structural relationships were estimated using bootstrapping with 5000 resamples (95% confidence intervals) to test the significance of path coefficients. The model’s explanatory power and predictive relevance were subsequently examined using the coefficient of determination (R^2^) and effect size (f^2^).

### 4.1. Measurement Model Assessment

The assessment results of the measurement model are summarized in Table 2. First, regarding indicator reliability, all standardized factor loadings ranged from 0.693 to 0.862. Although three items exhibited loadings marginally below the formally recommended 0.708 threshold—the level at which a construct explains more than 50 percent of the indicator’s variance—they were intentionally retained to preserve the theoretical content validity of the measurement scale. Specifically, within the Social Flow construct, item SF3 (loading = 0.693) captures the “effortless momentum” and dyadic synchrony; item SF2 (loading = 0.695) measures the “autotelic” (intrinsically rewarding) essence of the interaction; and item SF1 (loading = 0.710) indexes the core state of “cognitive absorption.” Deleting these specific indicators would severely compromise the conceptual breadth and ontological essence of the social flow construct. Statistically, their retention is fully justified by standard PLS-SEM guidelines (Hair et al., 2021), which dictate that indicators with outer loadings between 0.40 and 0.70 should only be removed if their deletion leads to an increase in Composite Reliability (CR) or Average Variance Extracted (AVE) above the required minimum thresholds. Given that the Social Flow construct’s CR (0.893) and AVE (0.544) already comfortably exceed the required 0.70 and 0.50 thresholds, respectively, the deletion of these theoretically vital items is neither statistically necessary nor conceptually advisable. Second, internal consistency reliability was established, with both Cronbach’s a (ranging from 0.799 to 0.859) and Composite Reliability (CR) values for all constructs exceeding the 0.70 threshold. Third, convergent validity was confirmed, as the Average Variance Extracted (AVE) values for all latent variables ranged from 0.544 to 0.721, well above the 0.50 cutoff that indicates a construct explains at least 50 percent of the variance of its items. Additionally, all Variance Inflation Factor (VIF) values were below 3.3, indicating that collinearity was not a critical issue at the indicator level, as ideally VIF values should be close to 3 and lower. For the second-order construct Calculative Subjectivity, we employed the repeated-indicators approach (Sarstedt et al., 2019) to establish validity. The paths from the second-order factor to its first-order dimensions—Extrinsic Merit and Performance Orientation—were positive and statistically significant, supporting the hierarchical factor structure. Moreover, the average variance extracted (AVE) for the higher-order construct exceeded 0.50, indicating satisfactory convergent validity at the second-order level.

### 4.2. Discriminant Validity

Discriminant validity was rigorously evaluated using two established criteria: the Fornell–Larcker criterion and the Heterotrait-Monotrait ratio of correlations (HTMT).

First, the Fornell–Larcker criterion posits that the square root of the Average Variance Extracted (AVE) for each construct must exceed its highest correlation with any other construct in the model. As presented in Table 3, the diagonal elements (representing the square roots of AVEs) were consistently higher than the off-diagonal elements (inter-construct correlations), thereby satisfying this requirement. This indicates that each construct accounts for more variance among its indicators than any other latent variable does.

Second, to ensure robustness, the HTMT criterion, considered superior to the Fornell–Larcker criterion for detecting a lack of discriminant validity in PLS-SEM, was examined. Analysis revealed that all HTMT values fell below the conservative threshold of 0.85 (Henseler et al., 2015). This confirms that the constructs are empirically distinct and do not exhibit conceptual overlap. Collectively, these results provide strong evidence of discriminant validity for the measurement model.

### 4.3. Structural Model Assessment and Hypothesis Testing

The overall quality of the structural model was assessed before the specific path analysis. First, global model fit was verified using the Standardized Root Mean Square Residual (SRMR). The SRMR value was 0.071, which is well below the conservative threshold of 0.08 (Henseler et al., 2015), indicating acceptable model fit. The analysis revealed an R^2^ value of 0.465 for the final dependent variable, Social Flow. This indicates that 46.5% of the variance is explained by calculative subjectivity and the mediating mechanisms it incorporates.

Furthermore, to assess out-of-sample predictive relevance, the PLSpredict procedure was employed (Shmueli et al., 2019). The $Q_{predict}^{2}$ values for all endogenous constructs were greater than zero, with the ultimate dependent variable, Social Flow, demonstrating robust predictive relevance ($Q_{predict}^{2}$ = 0.262). Additionally, effect sizes ($f^{2}$) were evaluated for all direct structural paths. The $f^{2}$ values ranged from 0.053 to 0.199, indicating small to moderate explanatory power across the specific structural linkages. Robustness was tested by including gender, age, education, and relationship status as control variables predicting social flow. The analysis confirmed no significant impact on social flow for age (β = 0.035, *t* = 0.812, *p* = 0.417), gender (β = −0.028, *t* = 0.654, *p* = 0.513), education (β = 0.015, *t* = 0.398, *p* = 0.691), or relationship status (β = −0.042, *t* = 1.025, *p* = 0.306). This exhaustive statistical transparency ensures that the core structural relationships remain robust regardless of demographic variations within the current sample parameters. To directly address the sample heterogeneity regarding relationship status (i.e., ongoing versus recalled relationships), we conducted supplementary subgroup analyses as a robustness check. Using PLS-SEM Multi-Group Analysis (PLS-MGA) (Henseler et al., 2009), the sample was stratified into a “currently partnered” group (comprising respondents *in a relationship* or *married*; *n* = 194) and a “retrospective recall” group (comprising *single/not dating* and *separated/divorced* respondents; *n* = 156). The ambiguous “single (dating)” category (*n* = 52) was deliberately excluded from this specific analysis to ensure a strict conceptual dichotomy. The MGA results revealed no statistically significant differences in any of the structural path coefficients between the two groups (all *p*-values for path differences > 0.05). Most importantly, when the structural model was estimated exclusively within the currently partnered subsample (*n* = 194), all hypothesized paths (H1–H5) remained statistically significant, with path coefficients closely mirroring the full-sample results. This confirms that our theoretical model is highly robust across both present and retrospective relational contexts, empirically mitigating concerns regarding recall bias.

To rigorously address potential conceptual overlap between the independent and dependent variables, a specific sensitivity analysis was conducted on the measurement of social flow. A theoretical review of the item wording suggested that two social flow indicators could potentially be interpreted as the conceptual inverse of calculative subjectivity, raising concerns of artifactual correlation. Consequently, we re-estimated the entire structural model after explicitly excluding these calculation-related items from the social flow construct. The results of this reduced-item sensitivity analysis demonstrated that all hypothesized structural paths (H1–H5) remained highly significant (*p* < 0.001), with path coefficients exhibiting only negligible deviations from the full-model estimates. This empirical confirmation robustly establishes that the substantive negative relationship between calculative subjectivity and social flow is driven by true relational dynamics rather than being an artifact built into their operationalization.

Hypothesis testing was subsequently conducted using Bootstrapping (5000 samples) on the full sample, as shown in Table 4. Additionally, a comprehensive summary of the structural model’s quality criteria, including $Q_{predict}^{2}$ and $f^{2}$, is detailed in Table 5.

The data analysis revealed a significant negative direct impact of calculative subjectivity on social flow (β = −0.214, *t* = 4.714, *p* < 0.001), supporting H1. This empirical finding supports the philosophical critique regarding the agony of eros (Han, 2017). It suggests that the immersive flow experience is directly weakened by instrumental rationality when intimate relationships are viewed as projects requiring input-output calculation.

Further, H2–H5 verified the specific pathways through which calculative subjectivity disrupts flow. Significant indirect effects were revealed for all four pathways, as confidence intervals excluded zero. Firstly, regret aversion demonstrated a substantial mediation effect (β = −0.088, *p* < 0.001), supporting H2. This result aligns with findings on maximizers, suggesting that the exhaustion of cognitive resources constitutes the primary barrier to flow experience, as calculative individuals are trapped in decision anxiety by excessive attention to potential superior options.

Secondly, sunk cost sensitivity played a significant mediating role (β = −0.064, *p* < 0.001), supporting H3. This aligns with the investment model. Irrational persistence is generated by an excessive accounting of past investments, such as time, money, and digital records, making it difficult for individuals to detach even from low-quality interactions.

Thirdly, a substantial adverse transmission effect was observed for the self-monitoring path (β = −0.081, *p* < 0.001), supporting H4. This finding corresponds with the theories of Snyder (1974) and Hall et al. (2010), suggesting that a genuine connection is blocked because the individual is unable to achieve de-individuation during interaction due to the strategic performance required to maintain an idealized self-presentation.

Finally, calculative subjectivity significantly reduced social flow through partner phubbing (β = −0.070, *p* < 0.001), supporting H5. Although the effect size is slightly smaller than that of cognitive variables, this finding corroborates Roberts and David’s (2016) view. A physical interruption of attention occurs when the forced digital presence required to assess value disrupts the continuity of dyadic interaction.

### 4.4. Importance-Performance Map Analysis (IPMA)

IPMA was conducted to further evaluate the practical priorities for intervention by examining the relative influence and prevalence of the antecedent variables on social flow. In PLS-SEM, IPMA typically evaluates positive total effects; however, because the constructs in this study act as inhibitors to intimacy, we adopted the absolute values of their negative total effects to represent their “disruptive importance”, that is, the magnitude of their detrimental impact on social flow. Average latent variable scores (rescaled to a 0–100 scale) were used as performance indices, reflecting the relative prevalence of each construct within the sample.

As detailed in Table 6, while the exogenous variable, calculative subjectivity, inherently exhibits the highest total disruptive effect (importance = 0.517) and highest prevalence (58.18) as the primary structural antecedent within the model, the most meaningful insights for relational intervention emerge from comparing the four mediating mechanisms. Among these mediators, regret aversion demonstrates the highest disruptive importance (0.217), closely followed by self-monitoring (0.206) and sunk cost sensitivity (0.191). Partner phubbing ranks lowest in importance among the mediators (0.187).

Conversely, the prevalence ranking (performance scores) reveals a different pattern. Among the mediators, partner phubbing is the most prevalent (57.16), followed by sunk cost sensitivity (55.70), regret aversion (54.72), and self-monitoring (54.22). When importance and performance are synthesized, a critical cognitive-behavioral paradox emerges: while behavioral manifestations such as partner phubbing are highly prevalent and observable, their actual structural potential to disrupt flow is the lowest among the mediators. Instead, interventions should prioritize the underlying cognitive and performative burdens—specifically regret aversion and self-monitoring—which pose the most severe threats to dyadic synchrony despite being slightly less outwardly prevalent.

## 5. Discussion and Implications

### 5.1. Discussion

The empirical findings substantiate the proposed model, demonstrating that calculative subjectivity functions as a potent disruptor of social flow in contemporary intimacy. By quantifying the interplay between internalized market logics and dyadic synchrony within a culturally involuted context, this study unveils how the “entrepreneurial self” navigates the paradoxes of digital romance under severe societal pressure. The significant negative association between calculative subjectivity and social flow (H1) provides empirical weight to Han (2017)’s philosophical lament on the “agony of eros,” yet goes beyond mere conceptualization by providing a quantifiable mechanism. While previous scholarship has identified the detrimental effects of extrinsic orientations on relationship satisfaction (Leung & Law, 2019; Unanue et al., 2014), our findings reveal a more fundamental ontological mismatch. Unlike individualistic flow studies that predominantly focus on skill-challenge balance (Csikszentmihalyi & Csikszentmihalyi, 1992), this study demonstrates that in a dyadic context, the instrumental, future-oriented nature of calculation is inherently antithetical to the autotelic, present-centered essence of flow (Walker, 2010). Crucially, this ontological mismatch is profoundly amplified by the specific cultural context of the Chinese sample. Within a hyper-competitive, “involuted” (neijuan) society, Western-originated neoliberal rationality intertwines with deeply ingrained traditional family expectations. In this environment, partner evaluation and relational optimization are rarely purely individualistic choices; rather, they are heavily burdened by collective parental pressures for marriage (e.g., cuihun) and societal mandates for material stability (e.g., housing and financial prerequisites). Consequently, the calculative subjectivity observed in Chinese digital intimacy is not merely an individual psychological defense, but a culturally mandated survival strategy where modern market logic and traditional familial obligations jointly colonize the autotelic essence of romance.

Crucially, the IPMA results offer a provocative counter-narrative to strictly technology-centric critiques (e.g., Cohen, 2019). Although partner phubbing (H5) remains a significant barrier, its relatively lower importance compared to cognitive mediators suggests that within the bounds of this specific model, the erosion of intimacy is statistically explained less by the “device in hand” (operationalized as phubbing behavior) and more by the “market in mind.” This implies that even in the physical absence of screens, the “calculative gaze” effectively colonizes the micro-moments of interaction, a finding broadly consistent with the theoretical interpretation that neoliberal governmentality has been internalized as a psychological imperative that thwarts autonomy and relatedness (Ginn et al., 2022; Ryan et al., 2021).

The prominent role of regret aversion as a key cognitive mediator (H2) illuminates the psychological toll of what Bauman (2013) terms “liquid” intimacy. Our results align with and extend maximization research in digital dating (Mikkelson & Ray, 2020; Pronk & Denissen, 2020), suggesting that the “shadow of the unchosen” precipitates a state of counterfactual rumination that depletes the cognitive resources necessary for social flow. While Ma and Roese (2014) focus on regret as a post-decisional outcome, our study repositions it as an ongoing, process-based disruptor. From a Self-Determination Theory perspective, this signals a unique form of competence frustration: the individual experiences reduced agency not due to a lack of choice, but due to an overwhelming algorithmic abundance that makes the confident affirmation of the current partner psychologically burdensome (Ryan et al., 2021).

Furthermore, the robust mediation of self-monitoring (H4) reflects the “authenticity paradox” inherent in neoliberal dating environments. Consistent with Hall et al. (2010) and J. Ward (2017), our data suggest that when individuals view themselves as “human capital” to be marketed, interaction shifts from spontaneous connection to defensive performance. However, while previous research focused on the link between self-monitoring and relationship conflict (Overall et al., 2015), our study highlights its role in inhibiting de-individuation. This performative preoccupation replaces mutual resonance with a sterile, self-surveilling consciousness, thereby thwarting the spontaneity required for dyadic synchrony (Wickham & Bond, 2020).

The role of sunk cost sensitivity (H3) further highlights a temporal entrapment that contrasts with traditional investment models. While Rusbult (1980) and Coleman (2009) emphasize how investments stabilize relationships by increasing commitment, our findings reveal a “dark side” regarding the quality of interaction. The obsessive auditing of past emotional outlays anchors the individual in a retrospective logic, fundamentally misaligned with the “effortless momentum” and present-focus of flow. This suggests that “rational” persistence, when associated with sunk cost sensitivity, may actually prevent the emotional resonance that social flow is intended to foster (Hackert et al., 2023).

Finally, the finding that partner phubbing (H5) serves as a significant but relatively weaker mediator positions it as a symptomatic manifestation rather than a fundamental structural antecedent. While this corroborates Roberts and David (2024)’s findings on digital interruptions, the lower IPMA importance score in our study contrasts with behavioral-focused research (e.g., Ni et al., 2025). We argue that these empirical results suggest phubbing can be theoretically interpreted as the physical extension of a calculative mindset, a tool for ongoing market surveillance and risk hedging (Aloia, 2023). This nuanced distinction implies that focusing solely on reducing phone use (McDaniel et al., 2021) may yield limited benefits if the underlying “calculative subjectivity” continues to fragment the attentional continuity required for flow.

### 5.2. Theoretical Implications

This research yields pivotal theoretical advancements by innovating across Self-Determination Theory, emotional capitalism, and flow theory within the specific context of neoliberal digital intimacy.

First, this study transcends the individualistic boundaries of SDT by integrating it with sociological critiques of neoliberal governmentality. Rather than treating extrinsic goals as mere personal value preferences (Kasser & Ryan, 1996; Ryan et al., 2021), we re-theorize them as internalized structural imperatives. By empirically validating how “calculative subjectivity” thwarts autonomy and relatedness via cognitive mediators, this model establishes a robust macro-micro linkage. This enables SDT to interrogate how structural forces—such as market logic and algorithmic governance—manifest as proximal psychological barriers (Foucault, 2010; Shahi, 2026). This shift from dispositional to socio-structural antecedents provides a vital framework for applying SDT in critiquing the systemic erosion of human well-being.

Second, the validation of calculative subjectivity as a second-order construct—synthesizing extrinsic merit and performance orientation—represents a significant measurement innovation for the study of “emotional capitalism” (Hu & Ge, 2025; Illouz, 2012). By synthesizing these dimensions, this integrated framework provides a cohesive theoretical structure to capture the complexities of neoliberal intimacy, advancing beyond the conceptual limitations of using fragmented constructs like materialism or exchange orientation. This innovation equips relationship science with a sophisticated tool to quantify the “instrumental turn” in intimacy, offering a scalable metric that can be extended to analyze the rationalization of social capital in professional and community spheres.

Third, this research achieves a conceptual leap in flow theory by positioning “social flow” as a fragile dyadic state vulnerable to calculative interference. Moving beyond traditional autotelic models focused on individual skill-challenge balance (Csikszentmihalyi & Csikszentmihalyi, 1992; Walker, 2010), we identify “instrumental consciousness” as a novel, systemic antagonist to dyadic synchrony. This ontological extension redefines social flow not merely as a high-quality experience, but as a behavioral barometer for resistance to neoliberal alienation and fragmentation.

Finally, our IPMA results offer a critical re-theorization of how specific technological behaviors interact with internal schemas. By demonstrating that internal calculative logic has a profound statistical association with disconnection that descriptively parallels and underpins physical partner phubbing, this study decenters the device—measured specifically in this model through the behavioral manifestation of phubbing—in digital relational scholarship. We propose a “Symptomatic Mediation” perspective: rather than acting as a universal, standalone technological determinant, device usage such as phubbing can serve as an interface for an already-internalized market rationality (Bozdağ, 2025; Cohen, 2019). This redirecting of the scholarly gaze from “screen-time” to “internalized logic” provides a new foundation for intervention designs in the era of surveillance capitalism.

### 5.3. Practical Implications

The empirical validation of the “market-in-mind” relative prominence over the “device-in-hand” necessitates a strategic pivot in relational interventions, prioritizing cognitive restructuring over mere behavioral modification. First, intervention strategies must target the internalized calculative logic that fuels relational erosion, specifically by addressing the maximization paradox inherent in digital intimacy. Recognizing that the pursuit of optimal relational ROI is strongly associated with profound dissatisfaction, individuals should be assisted through psychological counseling and relationship education to challenge their internal “entrepreneurial” framing of the Other. We recommend the promotion of a satisficing mindset, which encourages a shift in focus from the breadth of potential alternatives to the depth of present-centered engagement. By fostering a higher tolerance for relationship ambiguity and uncertainty—prerequisites for rebuilding social flow—individuals can begin to dismantle the defensive, risk-averse posture that thwarts dyadic synchrony.

Second, intervention strategies must transcend the simplistic logic of digital restriction or “digital detox” movements. Given the high prevalence yet relatively low disruptive importance of partner phubbing revealed by our IPMA results, mandatory technical disconnection often lacks long-term efficacy and may even be associated with withdrawal anxiety without addressing the underlying mechanisms. Ecologically valid interventions should instead be directed toward dissolving the deep-seated antecedents of digital distraction, namely the fear of missing out (FOMO) and the performance-monitoring pressures of surveillance capitalism. We advocate for a move toward “attentional hygiene,” where the focus shifts from eliminating the device to reclaiming the continuity of interaction. This involves training partners to recognize how algorithmic abundance triggers their calculative subjectivity, thereby empowering them to preserve a private sphere of non-instrumental, autotelic connection amid a totalizing digital market.

Third, these findings offer a critical foundation for the design of digital matchmaking platforms and broader public health policies. Rather than optimizing for “frictionless” swiping, platform developers should consider incorporating deliberate “design frictions” that discourage the commodity-like evaluation of partners and mitigate regret aversion. On a societal level, public health initiatives addressing the modern loneliness epidemic should integrate “digital relational literacy” into their curricula. By educating digital natives on how neoliberal market logics infiltrate their private desires, policymakers can support initiatives that protect the capacity for social flow as a vital component of social capital and public well-being.

### 5.4. Limitations and Future Research

Despite its contributions, this study entails several limitations that offer clear directions for future inquiry. First, the cross-sectional, single-source, and retrospective design precludes strict causal inference and introduces potential recall bias. Specifically, the potential endogeneity of partner phubbing must be acknowledged; reverse causality is highly plausible, as couples experiencing low social flow may resort to their smartphones (phubbing) as an escape mechanism from unrewarding interactions. Furthermore, while both procedural and statistical mitigations were employed, Common Method Bias (CMB) cannot be definitively eliminated. Future scholarship should employ longitudinal designs, multi-wave data collection (temporal separation), dyadic designs (surveying both partners simultaneously), or Experience Sampling Methods (ESM) to disentangle these bidirectional dynamics and capture the temporal evolution and real-time fluctuations of calculative subjectivity and social flow. Second, the sample’s concentration on young, highly educated Chinese digital natives limits generalizability. The neoliberal logic of calculation may manifest differently across varying socio-economic strata and collectivist cultures. Subsequent research should interrogate these mechanisms across diverse age cohorts and cultural contexts. Third, while Self-Determination Theory (SDT) was employed as an overarching interpretive framework, this study did not directly measure basic psychological need satisfaction or frustration. Similarly, while our theoretical framing draws heavily on sociological critiques of neoliberal governmentality and societal involution, these macro-level ideological forces were not directly measured as empirical variables. Consequently, our findings firmly establish statistical associations among the proximal psychological constructs, whereas the broader macro-structural internalization process remains a theoretically informed interpretation rather than a directly observed mechanism. Future research should incorporate validated SDT scales to empirically test the specific psychological pathways through which calculative subjectivity disrupts intimacy. Finally, the emerging frontier of human–AI intimacy represents a critical avenue for exploration. As emotional AI becomes ubiquitous, investigating whether algorithmic mediation fosters genuine dyadic synchrony or merely reinforces instrumental rationality will be essential for understanding connection in the digital age.

## 6. Conclusions

This study set out to investigate the relationship between neoliberal market logic and intimate relationships by explicitly addressing two core research questions. In response to the first question, our empirical findings reveal a robust statistical association, indicating that calculative subjectivity functions as a potent psychological barrier to social flow. When intimacy is framed through the instrumental lens of efficiency and return on investment, the spontaneous, present-centered experience of dyadic synchrony is fundamentally undermined. In response to the second question regarding the underlying mechanisms, this research reveals that this negative association is transmitted through a dual pathway of cognitive burden and behavioral interference. Crucially, the internal cognitive mechanisms—most notably regret aversion and self-monitoring—constitute the dominant mediating mechanisms of this disconnection, while behavioral manifestations like partner phubbing act as secondary symptoms of an overarching calculative mindset. Ultimately, this study concludes that restoring authentic intimacy in the digital age requires moving beyond superficial screen-time reductions to fundamentally deconstruct the deeply internalized “market-in-mind.”

## Figures and Tables

**Table 1:** Sample Demographics (*N* = 402).

Variable	Category	Frequency	Percentage
Gender	Female	230	57.2%
	Male	165	41.0%
	Other/Unspecified	7	1.8%
Age	18~25	112	27.9%
	26~30	138	34.3%
	31~40	87	21.6%
	41~50	43	10.7%
	50 above	22	5.5%
Education	High school or below	20	5.0%
	Associate degree	36	9.0%
	Bachelor	245	61%
	Master	81	20.1%
	Doctor	20	5.0%
Status	Single (Not dating)	128	31.8%
	Single (Dating)	52	12.9%
	In a relationship	105	26.1%
	Married	89	22.1%
	Separated/Devoice	28	7.0%

**Table 2:** Measurement Model Assessment.

		Factor Loading	Cronbach’s α	CR		AVE	VIF
				rho_c	rho_a		
CS	Extrinsic Merit						
	When judging a partner’s desirability, I place greater weight on income, social status, and physical attractiveness than on non-quantifiable traits.	0.781	0.846	0.886	0.847	0.564	2.091
	My sense of self-worth depends on how much my achievements and personal image are recognized by others.	0.752					2.070
	I tend to evaluate both myself and others using measurable markers such as earnings, credentials, rankings, or social visibility.	0.742					2.055
	Performance Orientation						
	I view intimate relationships as projects that require careful management and ongoing optimization toward measurable goals.	0.736					1.888
	When managing my relationship, outcomes I can track (e.g., goal attainment, efficiency) weigh more in my evaluations than the process itself.	0.742					2.089
	I actively manage externally visible indicators (e.g., public image, status signals) as part of optimizing relationship outcomes and returns.	0.754					1.952
RA	I always try to hold out for the “perfect” option out of fear that I might regret my choice later.	0.852	0.805	0.884	0.811	0.718	1.737
	My biggest fear is regretting it in the future when making decisions (about romance).	0.858					1.716
	It’s difficult for me to be delighted with my current partner because the infinite opportunities and illusory alternatives on dating apps make it hard.	0.833					1.761
SCS	It’s difficult for me to end this relationship because of the time and effort I have invested, which would be wasted.	0.841	0.799	0.882	0.802	0.713	1.705
	When I thought that my past emotional and material investments were for nothing, it was more painful than continuing to invest in the hope of better	0.833					1.683
	The accumulation of our digital memories (e.g., chat histories, photos) makes it harder for me to walk away from the relationship.	0.859					1.732
SM	I often act like very different people in different digital and dating situations to manage my image.	0.838	0.856	0.903	0.859	0.699	1.940
	I am good at observing others’ reactions (e.g., online feedback) and quickly adjusting my behavior to elicit the desired response.	0.835					2.014
	My social media profile is a curated version that shows only my best.	0.818					1.894
	I would adjust my (non-core) opinions to maintain a highly desirable image and avoid conflict with my partner.	0.851					2.062
PP	I always check my phone notifications involuntarily when I am with my partner.	0.862	0.806	0.886	0.808	0.721	1.854
	My phone is always put close to me, even when we are chatting.	0.844					1.648
	I check my phone whenever there is a brief lull or silence in our conversation	0.841					1.781
SF	I am fully absorbed in what we are doing when I am with my partner	0.710	0.859	0.893	0.860	0.544	1.907
	I do not weigh the potential payoffs because our interaction is its own reward.	0.695					1.752
	I don’t need to make a deliberate effort to keep them going because our conversations and activities always flow.	0.693					1.774
	I am free and relaxed about my performance when I am with him/her	0.732					1.937
	I can be my authentic self without having to second-guess or over-analyze what I say or do.	0.757					2.021
	I often keep my guard up, avoiding moments where I might completely lose myself in the interaction. (Reverse Coded)	0.785					2.226
	I often lose track of time when we are interacting deeply.	0.784					2.167

**Table 3:** Fornell–Larcker & Heterotrait-monotrait ratio (HTMT).

	Fornell–Larcker Criterion						Heterotrait-Monotrait Ratio (HTMT)				
	CS	RA	SCS	SM	PP	SF	CS	RA	SCS	SM	PP
CS	0.751										
RAO	0.408	0.848					0.488				
SCS	0.333	0.268	0.844				0.405	0.330			
SM	0.391	0.297	0.233	0.836			0.459	0.353	0.282		
DDS	0.376	0.277	0.222	0.306	0.849		0.453	0.341	0.273	0.367	
SF	−0.517	−0.468	−0.410	−0.456	−0.433	0.737	0.605	0.559	0.493	0.530	0.518

**Table 4:** Results of Hypothesis Testing.

	Path	β	T Value	*p* Value	Result
H1	CS → SF	−0.214	4.714	0.000	Support
H2	CS → RA → SF	−0.088	4.885	0.000	Support
H3	CS → SCS → SF	−0.064	4.071	0.000	Support
H4	CS → SM → SF	−0.081	4.188	0.000	Support
H5	CS → PP → SF	−0.070	4.024	0.000	Support

Note: CS = Calculative Subjectivity; SF = Social Flow; SCS = Sunk Cost Sensitivity; SM = Self-Monitoring; RA = Regret aversion; PP = Partner Phubbing.

**Table 5:** Structural Model Quality Assessment (Predictive Relevance and Effect Sizes).

Endogenous Construct	$Q_{predict}^{2}$	Direct Path	*f* ^2^	Effect Size Rating
Regret Aversion (RA)	0.161	CS → RA	0.199	Moderate
Self-Monitoring (SM)	0.148	CS → SM	0.180	Moderate
Partner Phubbing (PP)	0.133	CS → PP	0.164	Moderate
Sunk Cost Sensitivity (SCS)	0.105	CS → SCS	0.125	Small-to-Moderate
Social Flow (SF)	0.262	CS → SF	0.058	Small
		RA → SF	0.069	Small
		SM → SF	0.063	Small
		SCS → SF	0.058	Small
		PP → SF	0.053	Small

**Table 6:** Results of Importance-Performance Map Analysis (IPMA).

Construct	Total Effect	Performance (0–100)	Importance Ranking	Performance Ranking
Calculative Subjectivity (CS)	−0.517	58.18	1	1
Regret Aversion (RA)	−0.217	54.72	2	4
Self-Monitoring (SM)	−0.206	54.22	3	5
Sunk Cost Sensitivity (SCS)	−0.191	55.70	4	3
Partner Phubbing (PP)	−0.187	57.16	5	2

## Data Availability

Data are available from the corresponding author upon reasonable request.
